# Supplementary material for: New Insights into the Neuromyogenic Spectrum of a Gain of Function Mutation in SPTLC1
Source: Genes (Basel). 2022 May 17;13(5):893. doi: 10.3390/genes13050893 (PMC9140917; doi:10.3390/genes13050893)
Supplement: Supplementary file 1 [file genes-13-00893-s001.zip › genes-1610425-supplementary.pdf]

Supplementals:

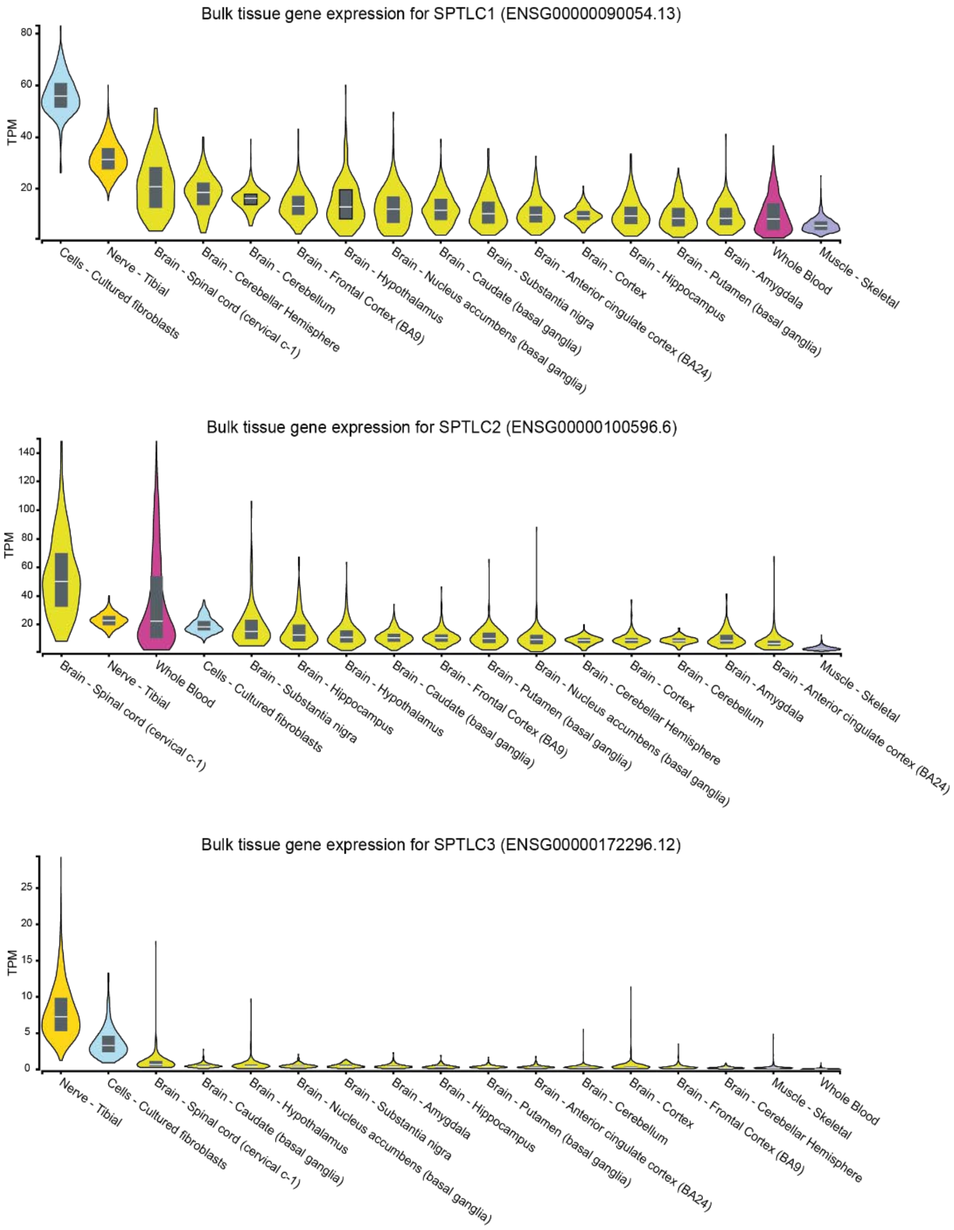

Figure S1: GTEx-based in silico analysis of tissue expression of *SPTLC1* (upper panel), ***SPTLC2***, (middle panel) **and *SPTLC3*** (lower panel). Expressed are log10-ratios of transcripts per million (TPM) in the respective tissues/nervous areas as violin plots. Order (from left to right) accords with expression level (from high to low).

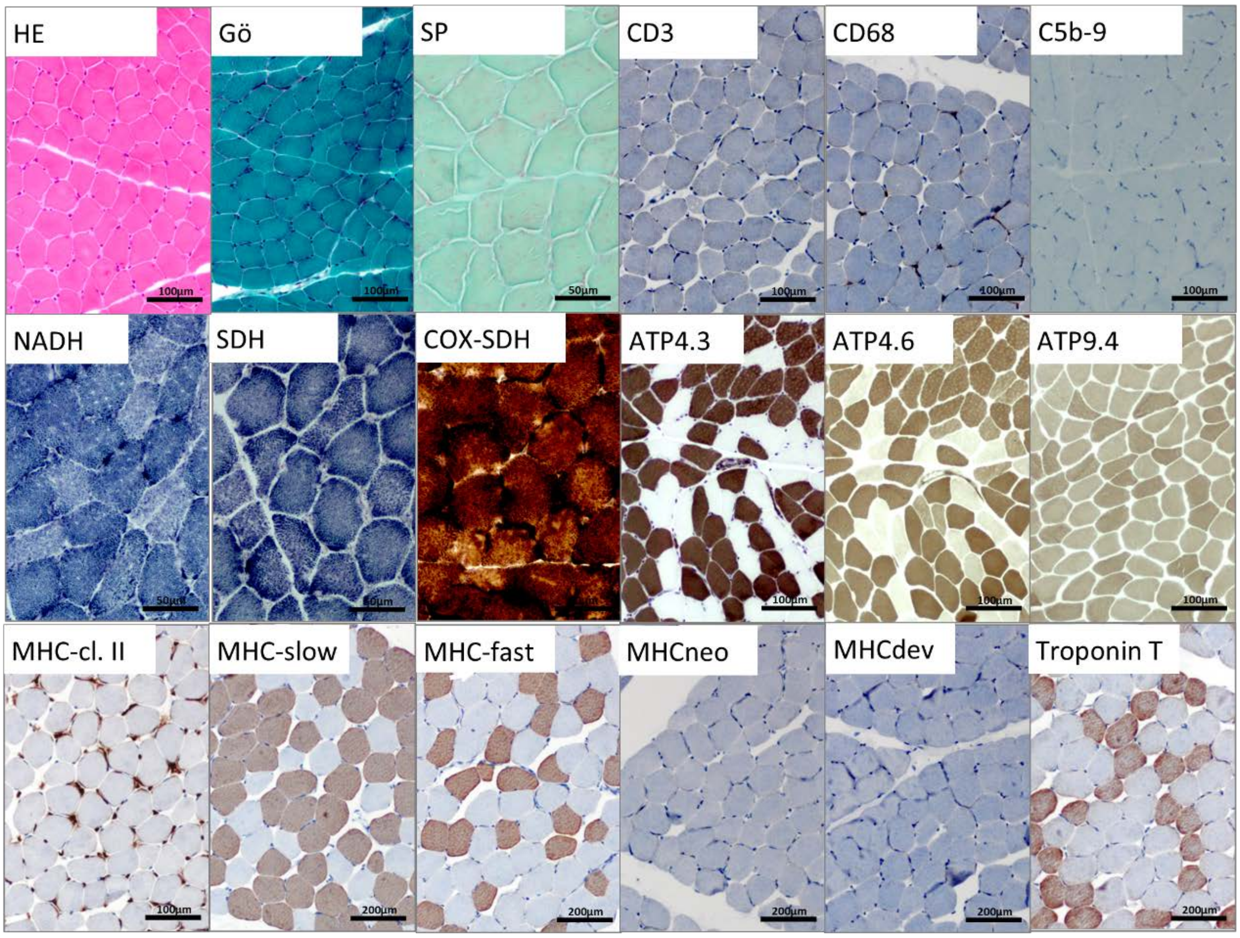

Figure S2: Staining of juvenile non-disease controls

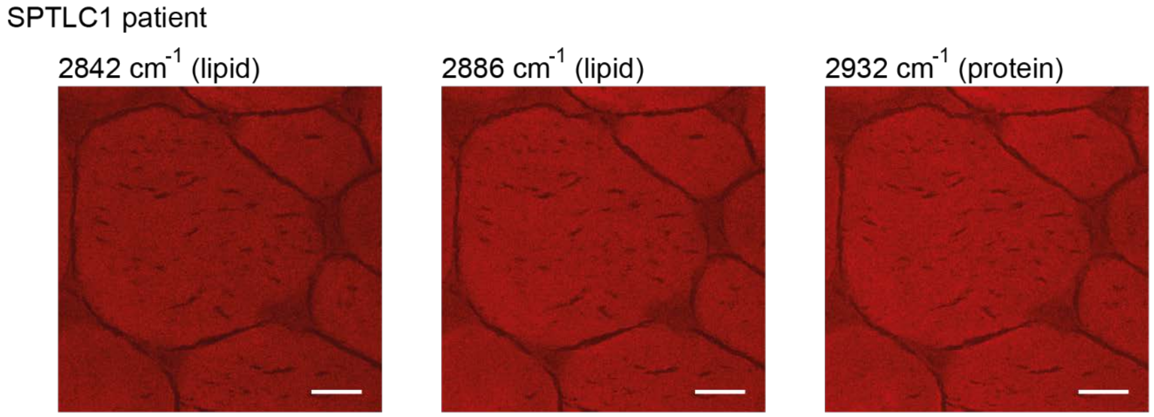

Figure S3. CARS images of the p.(A20S)-SPTLC1 patient-derived quadriceps muscle. No significant features could be detected at the wavenumbers 2842 cm^{-1} (lipid), 2886 cm^{-1} (lipid) and 2932 cm^{-1} (protein). (Scale bar: 15 μm).

Table S1

Accessions	Genes	Protein Descriptions	Unique Peptides	Fold Change	p Value
Q05397	PTK2	Focal adhesion kinase 1	3	13.10	0.034
P01876	IGHA1	Immunoglobulin heavy constant alpha 1	9	6.36	0.000
P01834	IGKC	Immunoglobulin kappa constant	5	3.90	0.000
P51452	DUSP3	Dual specificity protein phosphatase 3	2	2.86	0.006
Q03169	TNFAIP2	Tumor necrosis factor alpha-induced protein 2	4	2.82	0.020
P09417	QDPR	Dihydropteridine reductase	4	2.69	0.000
P22090	RPS4Y1	40S ribosomal protein S4, Y isoform 1	2	2.64	0.008
P30443	HLA-A	HLA class I histocompatibility antigen, A-1 alpha chain	7	2.61	0.002
P14151	SELL	L-selectin	3	2.26	0.007
O95816	BAG2	BAG family molecular chaperone regulator 2	2	2.02	0.001
P09913	IFIT2	Interferon-induced protein with tetratricopeptide repeats 2	2	1.99	0.005
Q96HC4	PDLIM5	PDZ and LIM domain protein 5	5	1.97	0.000
P28161	GSTM2	Glutathione S-transferase Mu 2	3	1.96	0.015
Q8N1S5	SLC39A11	Zinc transporter ZIP11	2	0.54	0.033
Q9HA77	CARS2	Probable cysteine--tRNA ligase, mitochondrial	9	0.54	0.000
Q9NVH6	TMLHE	Trimethyllysine dioxygenase, mitochondrial	2	0.54	0.004
Q9Y3L3	SH3BP1	SH3 domain-binding protein 1	9	0.53	0.034
P46934	NEDD4	E3 ubiquitin-protein ligase NEDD4	3	0.53	0.010
Q9UH65	SWAP70	Switch-associated protein 70	10	0.53	0.008
Q13423	NNT	NAD(P) transhydrogenase, mitochondrial	30	0.52	0.000
P16435	POR	NADPH--cytochrome P450 reductase	16	0.52	0.023
Q9ULJ6	ZMIZ1	Zinc finger MIZ domain-containing protein 1	2	0.51	0.019
Q14247	CTTN	Src substrate cortactin	6	0.51	0.047
Q6P179	ERAP2	Endoplasmic reticulum aminopeptidase 2	9	0.51	0.001
A8MXV4	NUDT19	Nucleoside diphosphate-linked moiety X motif 19	6	0.51	0.003
Q92643	PIGK	GPI-anchor transamidase	2	0.50	0.016
P82912	MRPS11	28S ribosomal protein S11, mitochondrial	3	0.50	0.008
P50416	CPT1A	Carnitine O-palmitoyltransferase 1, liver isoform	9	0.50	0.008
Q96S52	PIGS	GPI transamidase component PIG-S	5	0.49	0.002
P04792	HSPB1	Heat shock protein beta-1	13	0.49	0.052
P85037	FOXK1	Forkhead box protein K1	3	0.48	0.044
O15269	SPTLC1	Serine palmitoyltransferase 1	6	0.48	0.000
P21964	COMT	Catechol O-methyltransferase	3	0.46	0.032
Q92522	H1FX	Histone H1x	6	0.46	0.016
Q7Z4H8	KDELC2	KDEL motif-containing protein 2	2	0.46	0.025
P30837	ALDH1B1	Aldehyde dehydrogenase X, mitochondrial	7	0.46	0.002
Q9H7L9	SUDS3	Sin3 histone deacetylase corepressor complex component SDS3	2	0.45	0.023
P62072	TIMM10	Mitochondrial import inner membrane translocase subunit Tim10	2	0.44	0.038

P52429	DGKE	Diacylglycerol kinase epsilon	2	0.42	0.001
Q16643	DBN1	Drebrin	9	0.42	0.000
Q8IYQ7	THNSL1	Threonine synthase-like 1	2	0.41	0.002
P48595	SERPINB10	Serpin B10	8	0.41	0.005
Q8NFV4	ABHD11	Protein ABHD11	3	0.41	0.017
Q14571	ITPR2	Inositol 1,4,5-trisphosphate receptor type 2	9	0.41	0.019
P01871	IGHM	Immunoglobulin heavy constant mu	17	0.40	0.001
P08397	HMBS	Porphobilinogen deaminase	3	0.37	0.025
P26447	S100A4	Protein S100-A4	3	0.33	0.000
P34913	EPHX2	Bifunctional epoxide hydrolase 2	3	0.32	0.038
Q92597	NDRG1	Protein NDRG1	2	0.31	0.004
Q08623	PUDP	Pseudouridine-5'-phosphatase	2	0.29	0.003
Q15771	RAB30	Ras-related protein Rab-30	2	0.27	0.027
P80723	BASP1	Brain acid soluble protein 1	10	0.25	0.001
Q8TCB0	IFI44	Interferon-induced protein 44	2	0.23	0.001
Q6DN90	IQSEC1	IQ motif and SEC7 domain-containing protein 1	3	0.23	0.000
B9A064	IGLL5	Immunoglobulin lambda-like polypeptide 5	2	0.22	0.000
Q7L3T8	PARS2	Probable proline--tRNA ligase, mitochondrial	2	0.19	0.010
P80748	IGLV3-21	Immunoglobulin lambda variable 3–21	2	0.11	0.000
P04083	ANXA1	Annexin A1	10	0.10	0.000
P0DOY2	IGLC2;IGLC3	Immunoglobulin lambda constant 2;Immunoglobulin lambda constant 3	4	0.07	0.000
